# Reactivation of ERK and Akt confers resistance of mutant BRAF colon cancer cells to the HSP90 inhibitor AUY922

**DOI:** 10.18632/oncotarget.10414

**Published:** 2016-07-06

**Authors:** Chun Yan Wang, Su Tang Guo, Jia Yu Wang, Xu Guang Yan, Margaret Farrelly, Yuan Yuan Zhang, Fen Liu, Hamed Yari, Ting La, Fu Xi Lei, Lei Jin, Xu Dong Zhang, Chen Chen Jiang

**Affiliations:** ^1^ School of Biomedical Sciences and Pharmacy, The University of Newcastle, NSW, Australia; ^2^ Department of Molecular Biology, Shanxi Cancer Hospital and Institute, Taiyuan, Shanxi, China; ^3^ School of Medicine and Public Health, The University of Newcastle, NSW, Australia

**Keywords:** colon cancer, mutant BRAF, heat shock protein 90, AUY922, CDC37

## Abstract

Oncogenic mutations of BRAF occur in approximately 10% of colon cancers and are associated with their resistance to clinically available therapeutic drugs and poor prognosis of the patients. Here we report that colon cancer cells with mutant BRAF are also resistant to the heat shock protein 90 (HSP90) inhibitor AUY922, and that this is caused by rebound activation of ERK and Akt. Although AUY922 triggered rapid reduction in ERK and Akt activation in both wild-type and mutant BRAF colon cancer cells, activation of ERK and Akt rebounded shortly in the latter leading to resistance of the cells to AUY922-induced apoptosis. Reactivation of ERK was associated with the persistent expression of mutant BRAF, which, despite being a client of HSP90, was only partially degraded by AUY922, whereas reactivation of Akt was related to the activity of the HSP90 co-chaperone, cell division cycle 37 (CDC37), in that knockdown of CDC37 inhibited Akt reactivation in mutant colon cancer cells treated with AUY922. In support, as a HSP90 client protein, Akt was only diminished by AUY922 in wild-type but not mutant BRAF colon cancer cells. Collectively, these results reveal that reactivation of ERK and Akt associated respectively with the activity of mutant BRAF and CDC37 renders mutant BRAF colon cancer cells resistant to AUY922, with implications of co-targeting mutant BRAF and/or CDC37 and HSP90 in the treatment of mutant BRAF colon cancers.

## INTRODUCTION

Despite recent advances in the treatment of colon cancer, the overall survival of patients with metastatic colon cancers remains disappointing [1–3]. Although activating mutations of BRAF (BRAF^V600E^) are found only in a small proportion of colon cancers, this poses a great challenge in the quest for a better outcome of the patients, in that BRAF mutations, like mutations in KRAS, are associated with resistance of colon cancer to conventional chemotherapeutic drugs and agents targeting the epidermal growth factor receptor (EGFR) [4–6]. In addition, mutations in BRAF are associated with poor patient prognosis [6–8].

Heat shock protein 90 (HSP90) is one of the most abundant molecular chaperones and is essential for folding, stabilization and activation of a large number of proteins [9–11]. In particular, many mutant and overexpressed oncoproteins such as EGFR, CRAF and Akt are client proteins of HSP90 [11, 12]. As such, targeting HSP90 appears a promising approach in cancer treatment [12, 13]. Indeed, a number of HSP90 inhibitors such as AUY922 that do not significantly affect normal cell survival are currently in clinical studies for the treatment of various types of cancers including wild-type KRAS colon cancer [14–16]. Intriguingly, mutant BRAF is a client protein of HSP90, whereas HSP90-mediated chaperoning is not required for stabilization of wild-type BRAF [17, 18]. This suggests that HSP90 inhibitors may be of particular importance in the treatment of colon cancers carrying mutant BRAF.

HSP90-mediated chaperoning of proteins requires recruitment of clients through various co-chaperones [19–21], which function as scaffold proteins binding to HSP90 and its substrates simultaneously thus facilitating their interaction. Among HSP90 co-chaperones is cell division cycle 37 (CDC37) that interacts with a large proportion of protein kinases including cyclin-dependent kinases (CDKs), Akt and EGFR and thus directs their maturation [22–24]. Interestingly, CDC37 can also function as a protein chaperone independently of HSP90 [25, 26]. Nevertheless, it has been recently reported that although CDC37 can stabilize kinase clients independently of a direct interaction with HSP90, the activity of HSP90 is required [27]. It seems that the interrelating relationship between CDC37 and HSP90 in terms of protein chaperoning is highly dependent on client proteins in question. Indeed, CDC37 can compensate for HSP90 in maintaining some but not all protein clients [26]. Importantly, CDC37 itself has protein kinase activity that is important for phosphorylation of a number of its kinase clients [23, 28].

We have previously shown that the HSP90 inhibitor AUY922 preferentially induces apoptosis in colon cancer cells carrying mutant KRAS [29]. In this study, we have examined the impact of mutations in BRAF on the response of colon cancer cells to AUY922. We report here that mutant BRAF colon cancer cells are more resistant to AUY922 than those harboring wild-type BRAF due to rapid rebound activation of ERK and Akt. In addition, we demonstrate that reactivation of ERK is due to incomplete degradation of mutant BRAF by AUY922, whereas reactivation of Akt is triggered by the activity of CDC37 in mutant BRAF colon cancer cells.

## RESULTS

### Mutant BRAF colon cancer cells are resistant to AUY922

**Figure 1:**
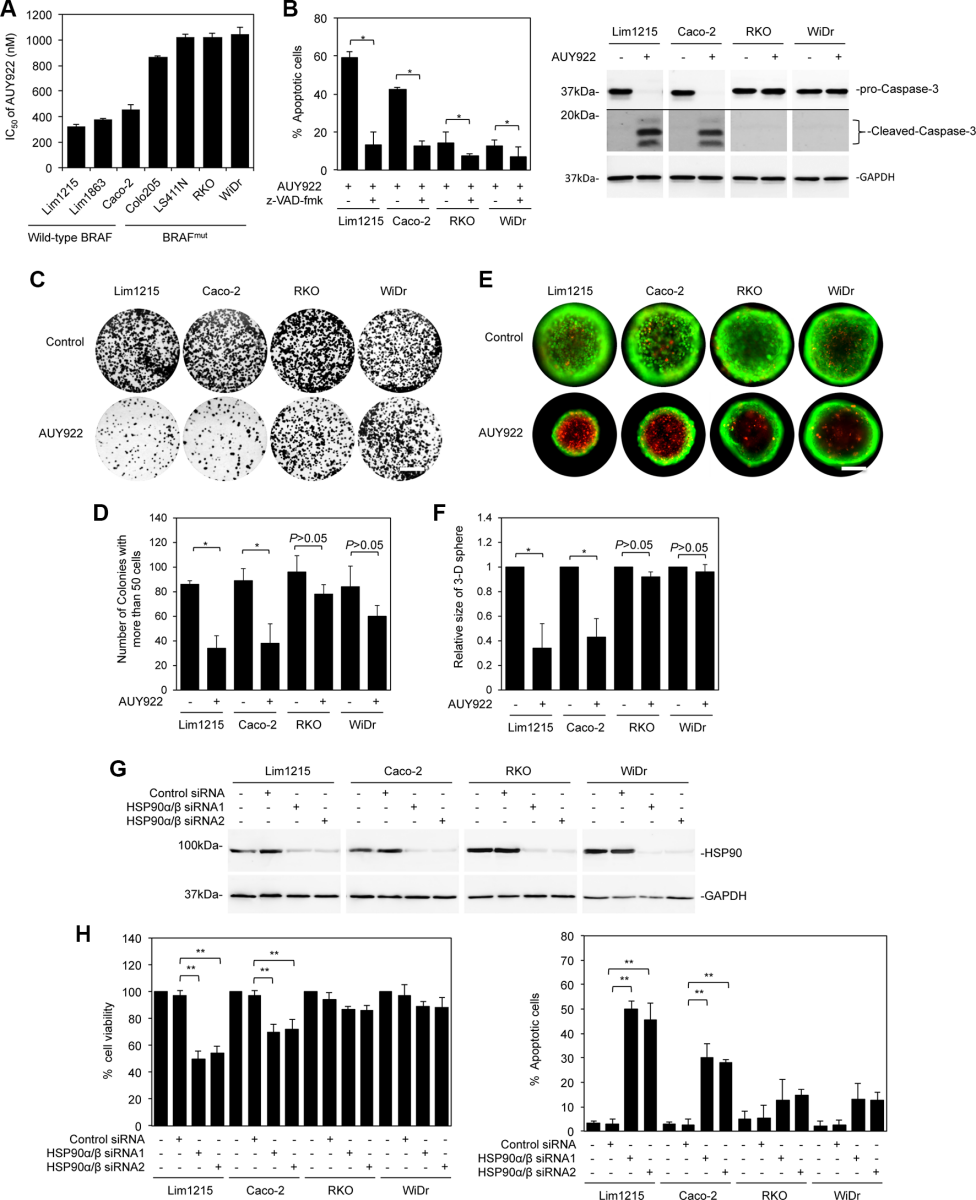
Mutant BRAF colon cancer cells are resistant to AUY922 (**A**) Comparison of IC_50_ of AUY922 in colon cancer cell lines treated with AUY922 for 48 hours. Data are mean ± SE, *n* = 3. (**B**) Cells were treated with z-VAD-fmk (30 μM) for 1 hour before adding AUY922 (400 nM) for 48 hours. Apoptosis was measured by PI and Annexin V staining (left panel). Whole cell lysates were subjected to Western blot analysis (right panel). Data are representative (right) or mean ± SE (left), *n* = 3. *P < 0.05, Student's *t*-test. (**C**) Cells seeded at 2000 cells/well onto 6-well plates were treated with AUY922 (400 nM). Twelve days later, cells were stained with crystal violet. Scale bar, 1cm. Data are representative, *n* = 3. (**D**) Quantitation of numbers of colonies as shown in Figure 1C. Data are mean ± SE, *n* = 3. *P < 0.05, Student's *t*-test. (**E**) Cells were seeded at 500 cells/well onto a 96-well Perfecta3-D^®^ Hanging drop plate. Five days later, cells were stained with calcein AM and ethidium homodimer-1 for 24 hours followed by treatment with AUY922 (400nM) for 48 hours, Scale bars, 25 μm. Data are representative, *n* = 3. (**F**) Relative sizes represented by relative diameters of colon cancer cell spheres as shown in Figure 1E. The diameter of the cell sphere treated with the vehicle control was arbitrarily designated as 1. Data are mean ± SE, *n* = 3. *P < 0.05, Student's *t*-test. (**G**) Cells were transfected with the control or HSP90α/β siRNAs were subjected to Western blot analysis. Data are representative, *n* = 3. (**H**) Cells were transfected with the control or HSP90α/β siRNAs were subjected to CellTiter-Glo assays and the PI and Annexin V staining. Data are mean ± SE, *n* = 3. **P < 0.01, Student's *t*-test.

We have previously shown that the HSP90 inhibitor AUY922 kills colon cancer cells through apoptosis and that wild-type KRAS colon cancer cells are more resistant to apoptosis induced by AUY922 compared with those carrying active mutations in KRAS [29]. In subsequent studies, we found that among wild-type KRAS colon cancer cells, those harbouring active mutations in BRAF were even more resistant to AUY922-induced apoptosis than cells with wild-type BRAF (Figure 1A and 1B). The half-maximum inhibitory concentration (IC_50_) values were > 800 nM in Colo205, LS411N, RKO and WiDr cells (mutant BRAF) compared with 326–458 nM in Lim1215, Lim1863, and Caco-2 cells (wild-type BRAF) (Figure 1A). The difference in the sensitivity of mutant compared to wild-type BRAF colon cancer cells to AUY922-induced apoptosis was also reflected in clonogenic assays and in cells grown in 3-dimensional cultures (Figure 1C–1F). Similar to AUY922, knockdown of the major isoforms of HSP90, HSP90α/β induced cell death in wild-type BRAF colon cancer cells, but did not significantly impinge on survival of mutant BRAF colon cancer cells (Figure 1G and 1H) [15, 30]. Moreover, mutant BRAF colon cancer cells were also relatively resistant to the other HSP90 inhibitor, XL888 (Supplementary Figure S1) [31].

### Resistance of mutant BRAF colon cancer cells to AUY922 is associated with rapid recovery of ERK and Akt activation

**Figure 2:**
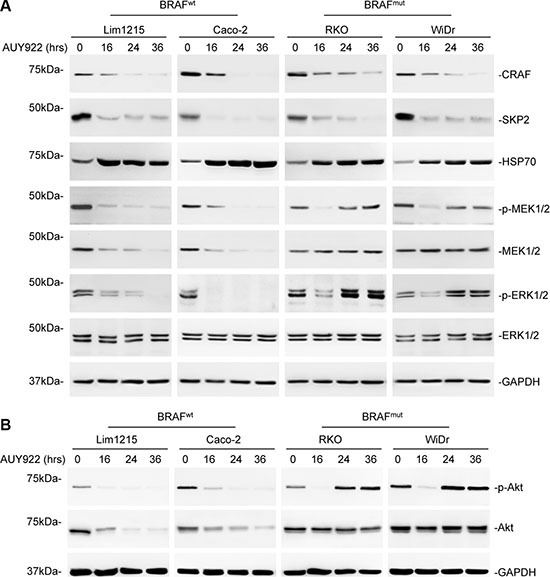
Resistance of mutant BRAF colon cancer cells to AUY922 is associated with rapid recovery of ERK and Akt activation (**A**) Whole cell lysates from Lim1215, Caco-2, RKO and WiDr cells treated with AUY922 (400 nM) for indicated time points were subjected to Western blot analysis. Data are representative, *n* = 3. (**B**) Whole cell lysates from Lim1215, Caco-2, RKO and WiDr treated with AUY922 (400 nM) for indicated time points were subjected to Western blot analysis. Data are representative, *n* = 3.

Since HSP90 is responsible for chaperoning of multiple protein kinases involved in activation of ERK and Akt including Akt itself, which are critical for cell survival [23, 32], we monitored the kinetics of ERK and Akt activation in colon cancer cells in response to AUY922. As anticipated, treatment with AUY922 resulted in marked reduction in activation of ERK and Akt in both wild-type (Lim1215 and Caco-2) and mutant (RKO and WiDr) BRAF colon cancer cells that was readily detectable at 16 hours (Figure 2A and 2B). This reduction was sustained for at least 36 hours after treatment in wild-type BRAF colon cancer cells (Figure 2A and 2B), but was diminished by 24 hours in mutant BRAF colon cancer cells (Figure 2A and 2B). The levels of ERK and Akt activation in mutant BRAF colon cancer cells at 24 and 36 hours after treatment were comparable to the basal levels observed in the cells before treatment (Figure 2A and 2B).

Of note, although Akt is a client protein of HSP90 [23, 33], AUY922 did not trigger any change in Akt expression in mutant BRAF colon cancer cells even at 16 hours when Akt activation was significantly suppressed (Figure 2B). In contrast, AUY922 markedly reduced Akt expression in wild-type BRAF colon cancer cells (Figure 2B). Therefore, the transient inhibitory effect of AUY922 on Akt activation in mutant BRAF colon cancer cells is primarily due to blockade of its upstream signals. Similarly, AUY922 did not alert the expression of MEK, another client protein of HSP90 [34], in mutant BRAF colon cancer cells (Figure 2A), whereas it reduced, albeit moderately, MEK expression in wild-type BRAF colon cancer cells (Figure 2A), suggesting that AUY922-induced transient inhibition of MEK/ERK activation in mutant BRAF colon cancer cells is also primarily due to blockade of upstream signals.

Despite its differential effects on Akt and ERK activation, AUY922 displayed otherwise comparable potency in inhibition of HSP90 in wild-type and mutant BRAF colon cells, as shown by similar degrees of reduction in its clients CRAF and S-phase kinase-associated protein 2 (SKP2) and upregulation of HSP70 induced by the inhibitor (Figure 2A) (29).

### Reactivation of ERK and Akt is responsible for resistance of mutant BRAF colon cancer cells to AUY922

**Figure 3:**
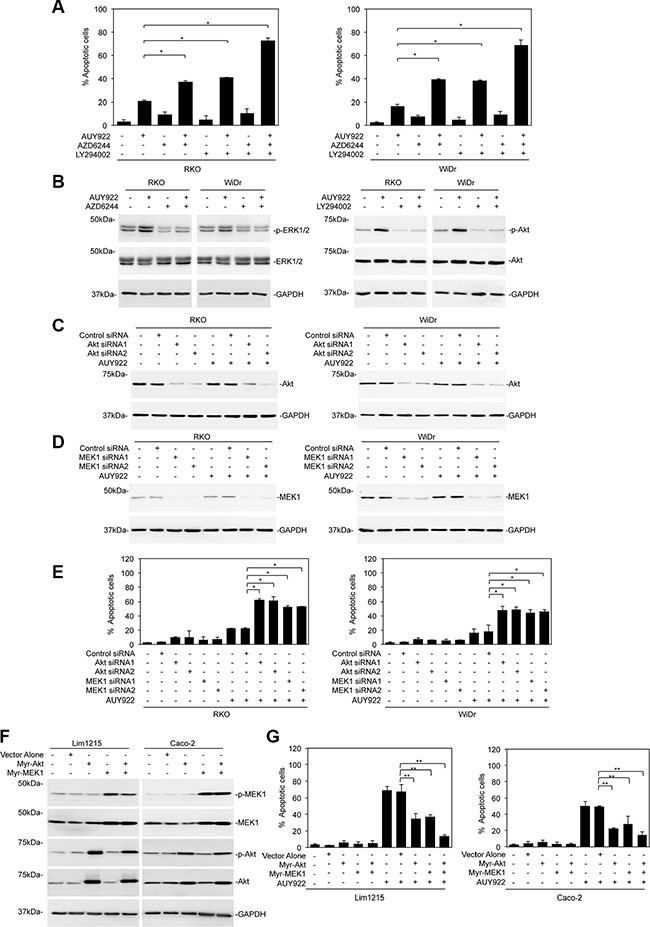
Reactivation of ERK and Akt is responsible for resistance of mutant BRAF colon cancer cells to AUY922 (**A**) RKO (left) and WiDr (right) cells were pre-treated with AZD6244 (1 μM) and LY294002 (40 μM) for 1 hour before addition of AUY922 (400 nM) for further 48 hours. Apoptosis was measured by PI and Annexin V staining. Data are mean ± SE, *n* = 3. *P < 0.05, Student's *t*-test. (**B**) Whole cell lysates from RKO and WiDr cells pre-treated with AZD6244 (1 μM) and LY294002 (40 μM) for 1 hour before addition of AUY922 (400 nM) for further 48 hours were subjected to Western blot analysis. Data are representative, *n* = 3. (**C**) Whole cell lysates from RKO (left) and WiDr (right) cells transfected with the control or Akt siRNAs were treated with AUY922 (400 nM) for 48 hours and subjected to Western blot analysis. Data are representative, *n* = 3. (**D**) Whole cell lysates from RKO (left) and WiDr (right) cells transfected with the control or MEK1 siRNAs were treated with AUY922 (400 nM) for 48 hours and subjected to Western blot analysis. Data are representative, *n* = 3. (**E**) RKO (left) and WiDr (right) cells transfected with the control, Akt or MEK1 siRNAs were treated with AUY922 (400 nM) for 48 hours. Apoptosis was measured by PI and Annexin V staining. Data are mean ± SE, *n* = 3. *P < 0.05, Student's *t*-test. (**F**) Whole cell lysates from Lim1215 and Caco-2 cells transduced with the control, myr-Akt or myr-MEK1 cDNA treated with AUY922 (400 nM) for 48 hours and subjected to Western blot analysis. Data are representative, *n* = 3. (**G**) Lim1215 (left) and Caco-2 (right) cells transduced with the control, myr-Akt or myr-MEK1 cDNA were treated with AUY922 (400 nM) for 48 hours. Apoptosis was measured by PI and Annexin V staining. Data are mean ± SE, *n* = 3. **P < 0.01, Student's *t*-test.

To examine the role of reactivation of ERK and Akt in resistance of mutant BRAF colon cancer cells to HSP90 inhibition, we treated RKO and WiDr cells with the MEK inhibitor AZD6244 or the PI3K inhibitor LY294002 before addition of AUY922. Indeed, although AZD6244 or LY294002 alone did not trigger significant cell death in RKO and WiDr cells, it sensitized the cells to AUY922-induced apoptosis (Figure 3A). This was associated with diminution of reactivation of ERK or Akt (Figure 3B). When AZD6244 and LY294002 were applied in combination, killing of mutant BRAF colon cancer cells by AUY922 was further enhanced (Figure 3A). Consistently, siRNA knockdown of MEK1 or Akt rendered RKO and WiDr cells sensitive to AUY922 (Figure 3C–3E). On the other hand, introduction of an active form of MEK1 (myr-MEK1) or an active form of Akt (myr-Akt) attenuated cell death induced by AUY922 in wild-type BRAF colon cancer cells [35, 36]. When myr-MEK1 and myr-Akt were co-introduced, AUY922-induced killing of Lim1215 and Caco-2 cells was abolished (Figure 3F and 3G). Together, these results indicate that reactivation of ERK and Akt plays an important role in resistance of mutant BRAF colon cancer cells to AUY922.

### Reactivation of ERK in colon cancer cells after treatment with AUY922 is due to the persistent expression of mutant BRAF

**Figure 4:**
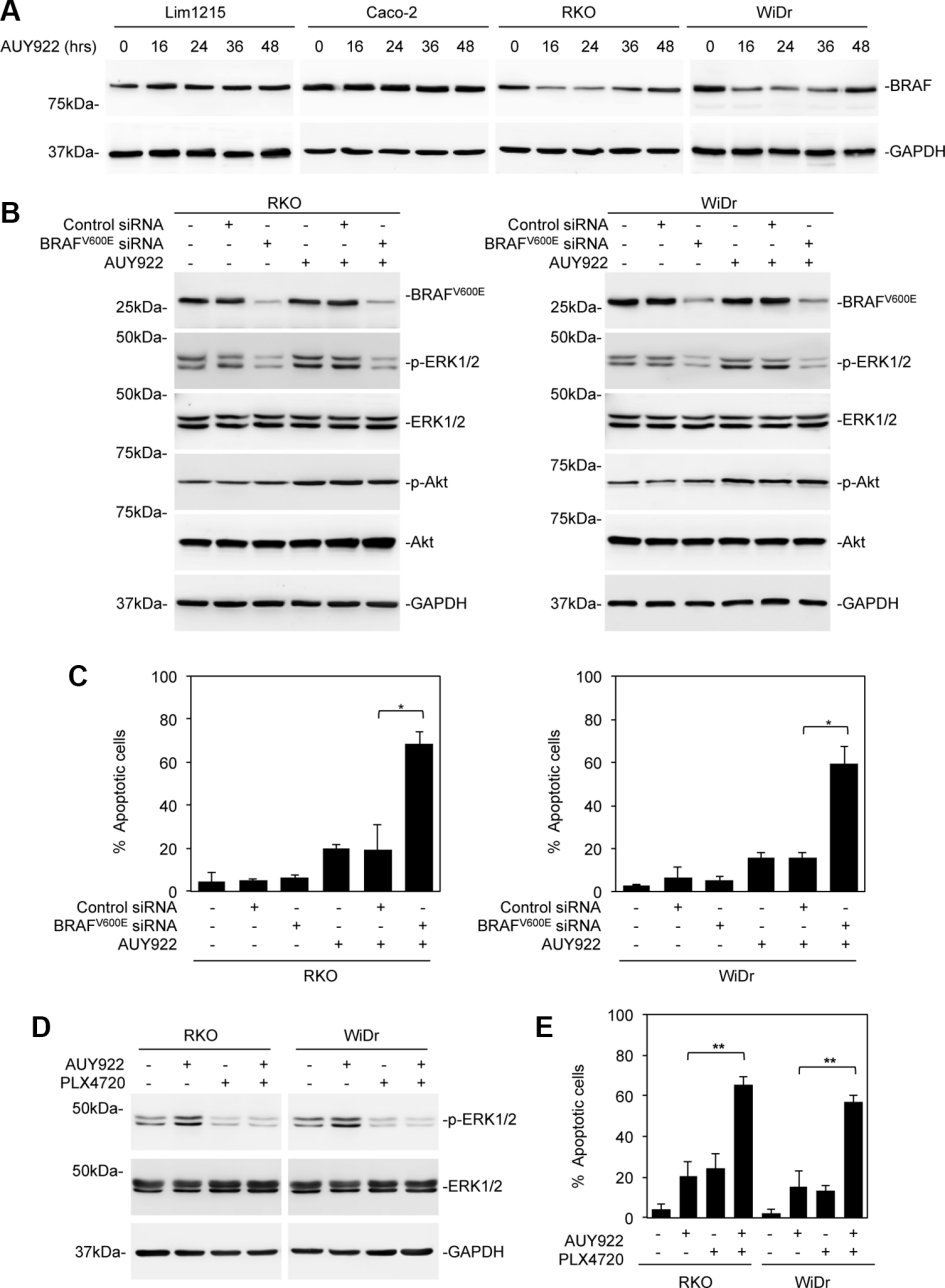
Reactivation of ERK in colon cancer cells after treatment with AUY922 is primarily due to persistent expression of mutant BRAF (**A**) Whole cell lysates from Lim1215, Caco-2, RKO and WiDr treated with AUY922 (400 nM) for indicated time points were subjected to Western blot analysis. Data are representative, *n* = 3. (**B**) RKO (left) and WiDr (right) cells transfected with the control or BRAF^V600E^ siRNA were treated with AUY922 (400nM) for 48 hours. Whole cell lysates were subjected to Western blot analysis. Data are representative, *n* = 3. (**C**) RKO (left) and WiDr (right) cells transfected with the control or BRAF^V600E^ siRNA were treated with AUY922 (400 nM) for 48 hours. Apoptosis was measured by PI and Annexin V staining. Data are mean ± SE, *n* = 3. *P < 0.05, Student's *t*-test. (**D**) Whole cell lysates from RKO and WiDr cells were treated with AUY922 (400 nM), and/or PLX4720 (3 μM) for 48 hours and subjected to Western blot analysis. Data are representative, *n* = 3. (**E**) RKO and WiDr cells were treated with AUY922 (400 nM), and/or PLX4720 (3 μM) for 48 hours. Apoptosis was measured by PI and Annexin V staining. Data are mean ± SE, *n* = 3. **P < 0.01 Student's *t*-test.

Although mutant BRAF is a client protein of HSP90 [17, 18], treatment with AUY922 caused only moderate reduction in mutant BRAF expression, whereas, as expected, it did not affect the expression of wild-type BRAF (Figure 4A). To examine whether remaining mutant BRAF (BRAF^V600E^) is involved in recovery of ERK activation in colon cancer cells after treatment with AUY922, we knocked down BRAF^V600E^ using a siRNA specific for the mutant form of BRAF (Figure 4B and 4C) [37]. Indeed, knockdown of BRAF^V600E^ conferred sensitivity of mutant BRAF colon cancer cells to AUY922-induced apoptosis (Figure 4C). This was associated with inhibition of constitutive activation of ERK and reactivation of ERK in cells after treatment with AUY922 (Figure 4B). Consistently, co-treatment with the mutant BRAF inhibitor PLX4720 inhibited reactivation of ERK and sensitized mutant BRAF colon cancer cells to AUY922-induced apoptosis (Figure 4D and 4E). On the other hand, BRAF^V600E^ knockdown did not impinge on reactivation of Akt (Figure 4B). These results suggest that the persistent expression of mutant BRAF is responsible, at least in part, for reactivation of ERK in mutant BRAF colon cancer cells after AUY922 treatment.

### CDC37 is responsible for reactivation of Akt in mutant BRAF colon cancer cells after treatment with AUY922

**Figure 5:**
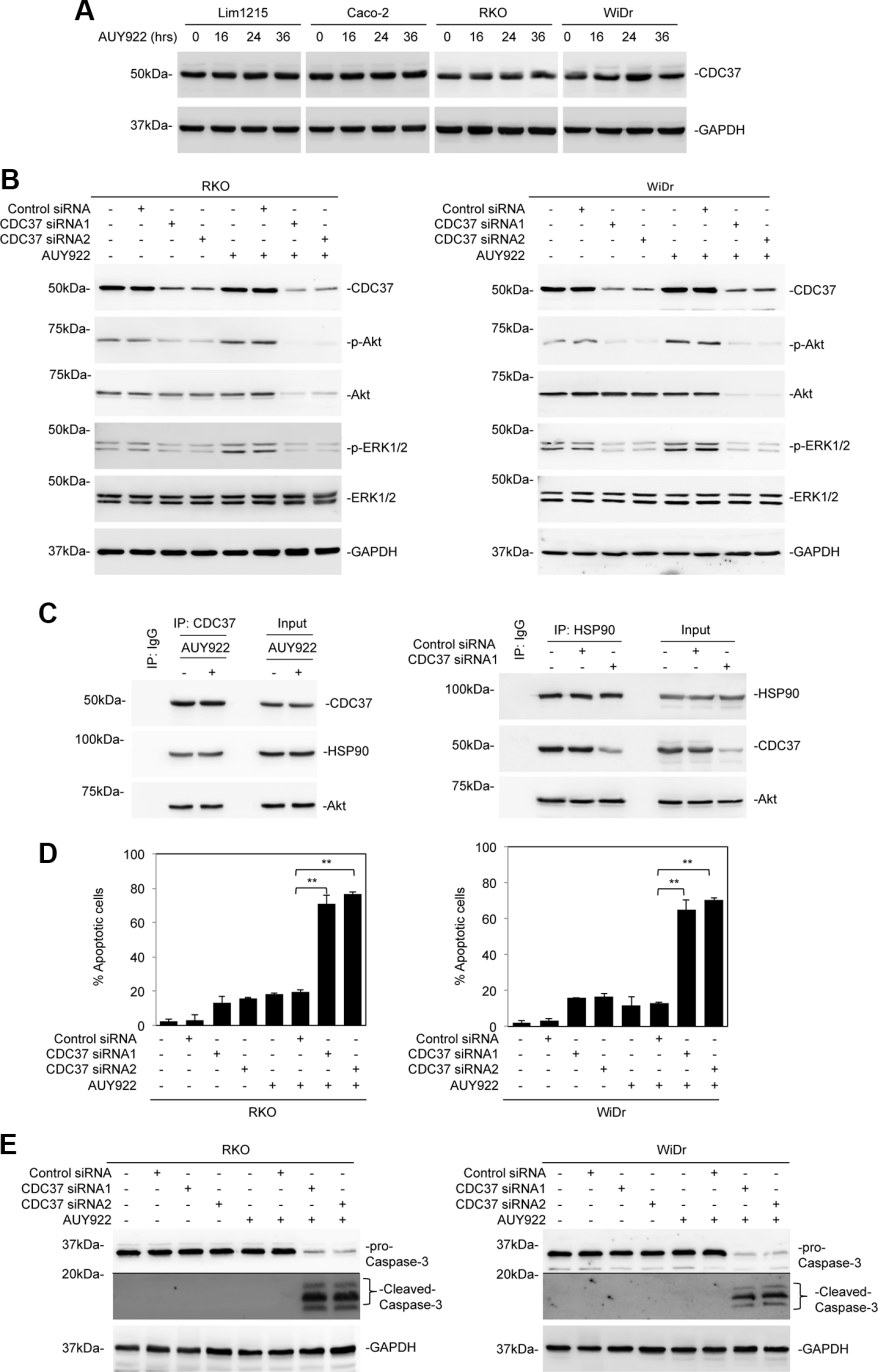
CDC37 is responsible for reactivation of Akt in mutant BRAF colon cancer cells after treatment with AUY922 (**A**) Whole cell lysates from Lim1215, Caco-2, RKO and WiDr treated with AUY922 (400 nM) for indicated time points were subjected to Western blot analysis. Data are representative, *n* = 3. (**B**) Whole cell lysates from RKO (left) and WiDr (right) cells transfected with the control or CDC37 siRNAs were treated with AUY922 (400 nM) for 48 hours and subjected to Western blot analysis. Data are representative, *n* = 3. (**C**) Left panel: Whole cell lysates from WiDr cells treated with AUY922 (400 nM) for 24 hours were subjected to immunoprecipitation with a mouse antibody against CDC37. The resulting precipitates were subjected to Western blot analysis. Right panel: Whole cell lysates from WiDr cells transfected with the control or CDC37 siRNA1 were subjected to immunoprecipitation with a rabbit antibody against HSP90. The resulting precipitates were subjected to western blot analysis. Data are representative, *n* = 3. (**D**) RKO (left) and WiDr (right) cells transfected with the control or CDC37 siRNAs were treated with AUY922 (400 nM) for 48 hours. Apoptosis was measured by PI and Annexin V staining. Data are mean ± SE, *n* = 3. **P < 0.01, Student's *t*-test. (**E**) Whole cell lysates from RKO (left) and WiDr (right) cells transfected with the control or CDC37 siRNAs were treated with AUY922 (400 nM) for 48 hours and subjected to Western blot analysis. Data are representative, *n* = 3.

Since CDC37 interacts with protein kinases and can stabilize and activate clients in concert with HSP90 or independently of HSP90 [23–26], we investigated whether CDC37 is involved in reactivation of Akt in mutant BRAF colon cancer cells treated with AUY922. Importantly, all the colon cancer cell lines included in this study harboured wild-type CDC37 as demonstrated by sequencing of all the 8 exons (including the intron/exon boundaries) of the gene. Moreover, CDC37 was expressed at comparable levels, which were not affected by treatment with AUY922, in the colon cancer cell lines irrespective of their mutational status of BRAF (Figure 5A and Supplementary Figure S2). Strikingly, siRNA knockdown of CDC37 not only inhibited rebound activation of Akt in mutant BRAF colon cancer cells after treatment with AUY922, but also reduced the basal levels of activation of Akt in these cells (Figure 5B). Nevertheless, it did not affect the expression of Akt in mutant BRAF colon cancer cells (Figure 5B), suggesting that CDC37 is not essential for chaperoning Akt in the cells. Of note, knockdown of CDC37 enabled AUY922 to diminish Akt expression (Figure 5B), indicating that either CDC37 or HSP90 is sufficient for stabilization of Akt, and that, when HSP90 is inhibited, CDC37 becomes essential for Akt stabilization and its subsequent activation. In support, CDC37 was co-precipitated with Akt in mutant BRAF colon cancer cells in the presence of AUY922, whereas HSP90 was similarly co-precipitated with Akt in these cells when CDC37 was knocked down (Figure 5C). Intriguingly, siRNA knockdown of CDC37 also reduced the basal levels of ERK activation, and inhibited its reactivation, albeit moderately, in mutant BRAF colon cancer cells after treatment with AUY922 (Figure 5B). Consistent with inhibition of reactivation of Akt and ERK, CDC37 knockdown sensitized mutant BRAF colon cancer cells to AUY922-induced apoptosis (Figure 5D and 5E).

### Mutant BRAF inhibitor, PI3K inhibitor, or knockdown of CDC37 overcomes resistance of colon cancer cells grown in 3-dimensional cultures to AUY922

**Figure 6:**
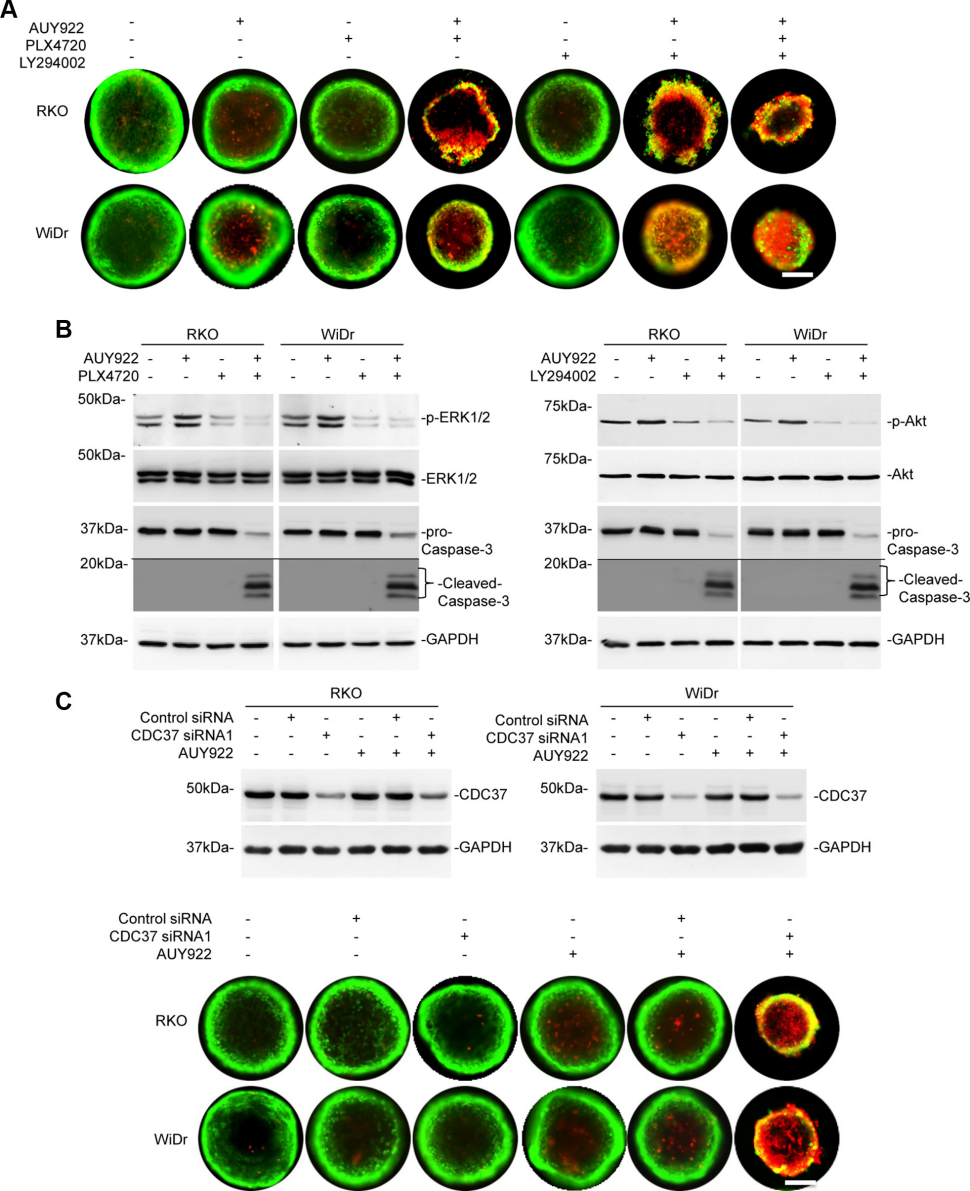
Mutant BRAF inhibitors, PI3K inhibitors, or knockdown of CDC37 overcomes resistance of colon cancer cells grown in 3-dimensional cultures to AUY922 (**A**) RKO and WiDr cells were seeded at 500 cells/well onto a 96-well Perfecta3-D^®^ Hanging drop plate. Five days later, cells were stained with calcein AM and ethidium homodimer-1 for 24 hours followed by treatment with AUY922 (400 nM) and/or PLX4720 (3 μM) and LY294002 (40 μM) for 48 hours. Data are representative, *n* = 3. Scale bars, 25 μm. (**B**) RKO and WiDr cells were seeded at 500 cells/well onto a 96-well Perfecta3-D^®^ Hanging drop plate. Five days later, cells were treated with AUY922 (400 nM) and/or PLX4720 (3 μM) (left) and LY294002 (40 μM) (right) for 48 hours. Whole cell lysates from 3-D spheres were subjected to Western blot analysis. Data are representative, *n* = 3. (**C**) Upper panel: RKO and WiDr cells transfected with the control or CDC37 siRNA1. Forty-eight hours later, five hundred cells were seeded onto a 96-well Perfecta3-D^®^ Hanging drop plate. After 5 days, cells were treated with AUY922 (400 nM) for 48 hours. Whole cell lysates from 3-D spheres were subjected to Western blot analysis. Data are representative, *n* = 3. Lower panel: RKO and WiDr cells transfected with the control or CDC37 siRNA1. Five hundred cells were seeded onto a 96-well Perfecta3-D^®^ Hanging drop plate. Five days later, cells were stained with calcein AM and ethidium homodimer-1 for 24 hours followed by treatment with AUY922 (400 nM) for 48 hours. Data are representative, *n* = 3. Scale bars, 25 μm.

To test the therapeutic potential of the finding that reactivation of ERK and Akt is responsible for resistance of mutant BRAF colon cancer cells to AUY922, we treated RKO and WiDr cells grown in 3-dimensional cultures with AUY922 in the presence or absence of the BRAF inhibitor PLX4720 or the PI3K inhibitor LY294002. While none of the compounds alone impinged on survival of the cells, co-treatment with PLX4720 or LY294002 and AUY922 triggered death of the cells in 3-dimensional cultures (Figures 6A, 6B and Supplementary Figure S3). The combination of PLX4720, LY294002, and AUY922 further enhanced killing of the cells (Figure 6A). As shown in Figure 6C, AUY922 induced cell death in RKO and WiDr cells with CDC37 knocked down grown in 3-dimensional cultures (Supplementary Figure S4).

## DISCUSSION

Although oncogenic mutations of BRAF are only seen in a small proportion of colon cancers, they pose a great challenge in the treatment of the disease, in that colon cancers with mutations in BRAF, similar to those with mutations in KRAS, are resistant to conventional chemotherapeutic drugs and agents targeting EGFR [4–6, 38]. Moreover, mutant BRAF colon cancer cells are largely unresponsive to clinically available mutant BRAF inhibitors including vemurafenib and dabrafenib, although these inhibitors have achieved impressive clinical response rates in the treatment of patients with mutant BRAF melanomas [39, 40]. In this report, we have presented evidence that colon cancer cells with mutations in BRAF are also resistant to HSP90 inhibitors, including AUY922 that is currently in clinical trials for the treatment of wild-type KRAS colon cancers [14]. In addition, we have shown that rebound activation of ERK and Akt caused respectively by the persistent expression of mutant BRAF and the activity of CDC37 is responsible for resistance of mutant BRAF colon cancer cells to AUY922.

HSP90 is responsible for chaperoning of a large number of oncogenic proteins that commonly converge on activation of ERK and Akt [32, 41, 42]. Indeed, inhibition of HSP90 by AUY922 resulted in rapid reduction in ERK and Akt activation in colon cancer cells. However, while inhibition of ERK and Akt activation persisted in wild-type BRAF colon cancer cells, it was rapidly reversed in colon cancer cells carrying mutant BRAF. This appeared to be responsible for resistant of mutant BRAF colon cancer cells to AUY922, in that blockade of ERK or Akt activation pharmacologically or genetically sensitized the cells to AUY922. On the other hand, introduction of active form of MEK1 or Akt rendered wild-type BRAF colon cancer cells resistant to AUY922. These results indicate that AUY922 and conceivably other HSP90 inhibitors are unlikely to achieve significant therapeutic responses in mutant BRAF colon cancers unless used in combination with inhibitors against MEK/ERK or/and PI3K/Akt.

Since mutant BRAF has been demonstrated to be a client of HSP90 [17, 18], it seems convincible that HSP90 inhibition would cause degradation of mutant BRAF and thus has the similar effect as a mutant BRAF inhibitor on mutant BRAF cells. However, to our surprise, treatment with AUY922 only moderately reduced the expression of mutant BRAF while it abolished the expression of other HSP90 client proteins such as CRAF and SKP2 in mutant BRAF colon cancer cells [29]. This suggests that mutant BRAF is less dependent on HSP90 for its stabilization than other HSP90 client proteins in colon cancer cells. Indeed, the persistent expression of mutant BRAF was responsible for rebound activation of ERK, as the addition of a BRAF^V600E^ inhibitor or specific knockdown of mutant BRAF inhibited reactivation of ERK after treatment with AUY922. However, why HSP90 inhibition cannot efficiently degrade mutant BRAF in colon cancer cells remains to be clarified. It is known that CDC37 is involved in HSP90-mediated chaperoning of mutant BRAF [18]. Intriguingly, our results showed that knockdown of CDC37 caused a moderate decrease in the basal levels of ERK activation and reactivation of ERK after treatment with AUY922, suggesting that CDC37 is able to stabilize mutant BRAF independently of HSP90 in colon cancer cells [23]. Regardless, our results suggest that the combination of mutant BRAF inhibitors and HSP90 inhibitors is a useful strategy to improve their therapeutic efficacy in the treatment of mutant BRAF colon cancer. Of note, inhibition of HSP90 by the inhibitor XL888 has been reported to potently sensitize mutant BRAF melanoma cells to BRAF inhibitors through diverse mechanisms [43]. It is conceivable that the inhibitory effect of AUY922 on multiple protein kinases such as CRAF and EGFR would also contribute the combinatorial effect of HSP90 inhibitors and BRAF inhibitors in colon cancer cells [44].

As a co-chaperone of HSP90, CDC37 is of particular importance for stabilization of protein kinases as it predominantly acts as a kinase-specific co-chaperone through bridging the physical association between HSP90 and client kinases before progression of the chaperone cycle [22, 45]. However, it is intriguing that neither inhibition of HSP90 by AUY922 nor knockdown of CDC37 caused reduction in the expression of Akt that is a client of HSP90 in mutant BRAF colon cancer cells. In contrast, when HSP90 and CDC37 were inhibited simultaneously, the levels of Akt expression were markedly decreased. These results indicate that either HSP90 or CDC37 alone is sufficient for stabilization of Akt in mutant BRAF colon cancer cells. In support, CDC37 was associated with Akt in the presence of AUY922, whereas HSP90 remained bound to Akt when CDC37 was knocked down. HSP90-independent chaperoning of proteins by CDC37 has been previously reported [26, 46]. Nevertheless, CDC37 did not appear to play a role in wild-type BRAF colon cancer cells, in that AUY922 alone inhibited Akt expression in the cells. Although how CDC37 acts differently on Akt in mutant and wild-type BRAF colon cancer cells remains unknown, it is unlikely caused by mutations in its gene as all the colon cancer cell lines included in this study harbored wild-type CDC37. Moreover, it is not related to differences in the expression of the protein, in that CDC37 was expressed at comparable levels between colon cancer cell lines carrying wild-type BRAF and whose with mutant BRAF. Similarly, it also remains to be determined how HSP90 interacts with Akt in mutant BRAF colon cancer cells deficient in CDC37. Regardless, our results clearly demonstrated that CDC37 plays an essential for rebound activation of Akt in mutant BRAF colon cancer cells after treatment with AUY922.

An important finding of this study was that CDC37 knockdown not only blocked rebound activation of Akt in mutant BRAF colon cancer cells after treatment with AUY922, but also inhibited constitutive activation of Akt in mutant but not wild-type BRAF colon cancer cells. In the absence of a change in the expression levels of Akt, these results indicate that CDC37 plays an important role in phosphorylation of Akt in mutant BRAF colon cancer cells. Although whether this is due to a direct phosphorylating effect of CDC37 on Akt or caused by activation of upstream protein kinases that regulates phosphorylation of Akt remains unclear, it is known that CDC37 can act as a protein kinase to phosphorylate its kinases clients [23, 28]. The effect of CDC37 on Akt phosphorylation, similar to its stabilizing effect on Akt, appears to be specific to colon cancer cells with activating mutations in BRAF, suggesting that mutant BRAF has reprogrammed the signaling network of protein kinases in colon cancer cells. Whether CDC37 has comparable effects on other kinases in mutant BRAF colon cancer cells and whether it has similar effects on other types of cells carrying mutations in BRAF needs further investigation. Our results suggest that CDC37 is a potential therapeutic target in the treatment of mutant BRAF colon cancers, in particular, in combination with HSP90 inhibitors.

The practical implications of the finding that rebound activation of ERK and Akt is responsible for resistance of mutant BRAF colon cancer cells to AUY922 was confirmed by using cells grown in 3-dimensional cultures. Co-treatment with a BRAF inhibitor or a PI3K inhibitor and AUY922 resulted in destruction of the tumor spheres. In addition, AUY922 triggered killing of mutant BRAF colon cancer cells with CDC37 knocked down in 3-dimensional cultures. These results may reflect more closely the response of mutant BRAF colon cells to the combinatorial approaches, and warrant further preclinical investigation of the combinations in the treatment of colon cancers with mutations in BRAF.

## MATERIALS AND METHODS

### Cell culture

Human colon cancer cell lines were described previously [47, 48]. Individual cell line authentication was regularly confirmed every 6 months using the AmpFISTR Identifiler PCR Amplification Kit from Applied Biosystems (Mulgrave, VIC, Australia) and GeneMarker V1.91 software (SoftGenetics LLC, State College, PA, USA). The last test was performed in November 2015.

### Antibodies (Abs) and reagents

Antibodies against HSP70 (#4872), CRAF (#9244), HSP90 (#4874), SKP2 (#2652), p-Akt (Ser473) (#4060), Akt (#9272), ERK1/2 (#9102), p-MEK1/2 (#9121), MEK1/2 (#9122), MEK1 (#9124) and p-MEK1 (#9127) were purchased from Cell Signalling Technology (Beverly, MA). Antibodies against p-ERK1/2 (sc-7383), BRAF (sc-166), CDC37 (sc-1617 and sc-13129) and GAPDH (sc-32233) were purchased from Santa Cruz Biotechnology (Santa Cruz, CA). The antibodies against Caspase-3 (AAP-113) were from Enzo Life Sciences (Dural, NSW, Australia). Antibody against BRAF^V600E^ (26039) was purchased from NewEast Biosciences (NewEast Biosciences, PA). The HSP90 inhibitors AUY922 and XL888, the MEK inhibitor AZD6244, the PI3K/Akt inhibitor LY294002 and the BRAF inhibitor PLX4720 were purchased from Selleckchem (Redfern, NSW, Australia).

### Cell viability

Cell viability was quantitated using the CellTiter-Glo Luminescent Cell Viability Assay Kit (Promega, San Luis Obispo, CA) as described previously [29]. Luminescence was recorded by Synergy 2 multi-detection microplate reader (BioTek, VT).

### Propidium iodide and Annexin V staining

Staining with Annexin V and Propidium iodide was carried out as described elsewhere [29]. In brief, cells were collected, washed twice with cold PBS and re-suspended in Annexin V binding buffer. Cells were then incubated with FITC-conjugated Annexin V and PE-conjugated Propidium iodide for 15 min in the dark followed by addition of binding buffer. Cells were analysed by flow cytometry within 1 h.

### Clonogenic assays

Clonogenic assays were carried out as described previously [29]. In brief, cells were seeded at 2000 cells/well onto 6-well culture plates. Cells were then allowed to grow for a further 12 days before fixation with methanol and staining with crystal violet (0.5% solution). Colonies with 50 or more cells were counted under a phase contrast microscope.

### Three-dimensional (3-D) culture

3-D culture was performed using the hanging drop technique as previously described [29]. Briefly, 500 cells were seeded onto the Perfecta3-D^®^ hanging drop plate (3-D Biomatrix, Ann Arbor, MI), and monitored with the Axiovert and Axioplan microscope (Carl Zeiss, North Ryde, NSW, Australia) for at least 5 days. Cells were then stained with calcein AM and ethidium homodimer-1 (Life Technologies, Scoresby, VIC, Australia) for 24 h followed by treatment. Spheres were harvested onto slides, and examined with a fluorescence microscope (Carl Zeiss). Diameters of cell spheres were quantified by image analysis using ImageJ software (NIH, Bethesda, MD) [49].

### Lentiviral gene transduction and DNA constructs

The myr-Akt and the myr-MEK1 cDNAs were cloned into the lentiviral expression plasmid pCDH-CMV-MCS-EF1-copGFP (Integrated Sciences, Chatswood, NSW, Australia). Lentiviral packaging was carried out as described previously [50]. Transduction efficiency was monitored by detecting green fluorescent protein (GFP) via flow cytometry.

### siRNA transfection

siRNAs against CDC37, Akt, and BRAF^V600E^ were synthesized by GenePharma (Shanghai GenePharma Co, Ltd, Shanghai, China) with the following target sequences: ACACAAGACCUUCGUGGAA (CDC37 siRNA1), CGGCAGUUCUUCACUAAGA (CDC37 siRNA2), GCUCCUUCAUUGGGUACAATT (Akt siRNA1), GCGGAAGGAAGUCAUCAUUTT (Akt siRNA2), GCUACAGAGAAAUCUCGAUTT (BRAF^V600E^ siRNA) [37]. MEK1 and HSP90α/β siRNAs were obtained from Dharmacon (Dharmacon, Lafayette, CO, USA) (MEK1 siRNA1 (M-003571-01-0005), MEK1 siRNA2 (D-003571-02-0002), HSP90α/β siRNA1 (M-005186-06) and HSP90α/β siRNA2 (M-005186-07)). Transfection of siRNA was carried out as described previously [29].

### Western blotting

Western blot was carried out as described previously [51]. Labeled bands were detected by Immun-Star^TM^ HRP Chemiluminescent Kit, and images were captured and the intensity of the bands was quantitated with the Bio-Rad VersaDoc^TM^ image system (Bio-Rad, Regents Park, NSW, Australia).

### Immunoprecipitation

Immunoprecipitation was carried out as described previously [35]. Briefly, whole cell lysates were incubated with the primary antibody or corresponding IgG for 2 h at 4°C, followed by incubation with 50 μl of protein A/G beads (Thermo Fisher Scientific, Scoresby, VIC, Australia). The bound proteins were eluted and processed for Western blot analysis. Antibodies used for immunoprecipitation were CDC37 (sc-13129) from Santa Cruz and HSP90 [3G3] (ab5457) from Abcam (Abcam plc, Melbourne, VIC, Australia).

### Statistical analysis and data presentation

Statistical analysis was performed using JMP Statistics Made Visual software. Student's *t*-test was used to assess differences between different groups. A *P* value less than 0.05 was considered statistically significant.

## SUPPLEMENTARY MATERIALS FIGURES



## Abbreviations

HSP90: heat shock protein 90
CDC37: cell division cycle 37
EGFR: epidermal growth factor receptor
CDKs: cyclin-dependent kinases
SKP2: S-phase kinase-associated protein 2
IC^50^: the half-maximum inhibitory concentration.
