# Supplementary material for: Reactivation of ERK and Akt confers resistance of mutant BRAF colon cancer cells to the HSP90 inhibitor AUY922
Source: Oncotarget. 2016 Jul 6;7(31):49597–610. doi: 10.18632/oncotarget.10414 (PMC5226532; doi:10.18632/oncotarget.10414)
Supplement: Supplementary file 1 [file oncotarget-07-49597-s001.pdf]

Reactivation of ERK and Akt confers resistance of mutant BRAF colon cancer cells to the HSP90 inhibitor AUY922

Supplementary Materials

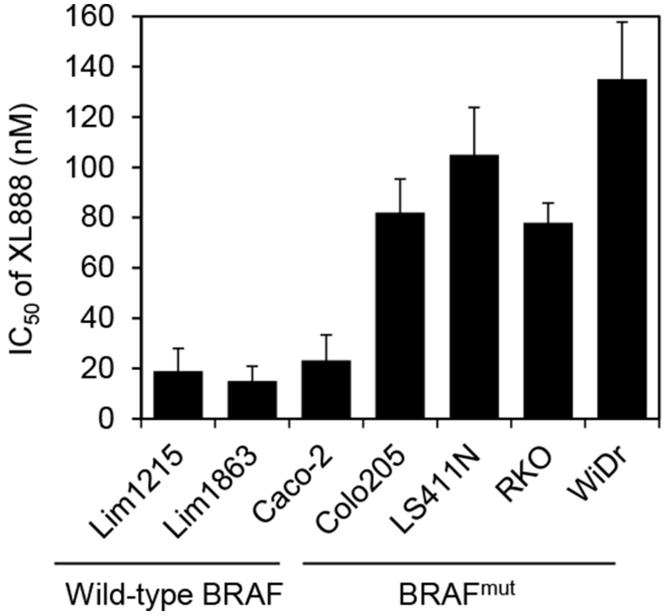

Supplementary Figure S1: Mutant BRAF colon cancer cells are resistant to XL888. IC_{50} values of XL888 in colon cancer cell lines treated with XL888 for 48 hours. Data are mean ± SE, $n = 3$.

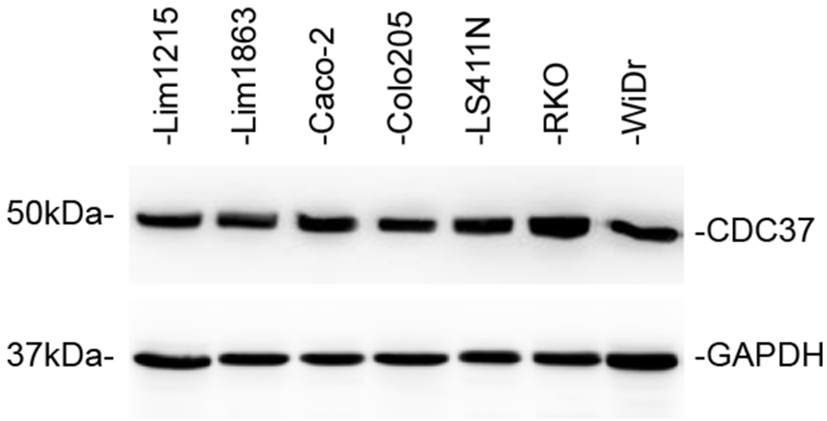

Supplementary Figure S2: The expression level of CDC37 in a panel of colon cancer cell lines. Whole cell lysates from Lim1215, Lim1863, Caco-2, Colo205, LS411N, RKO and WiDr cells were subjected to Western blot analysis. Data are representative, *n* = 3.

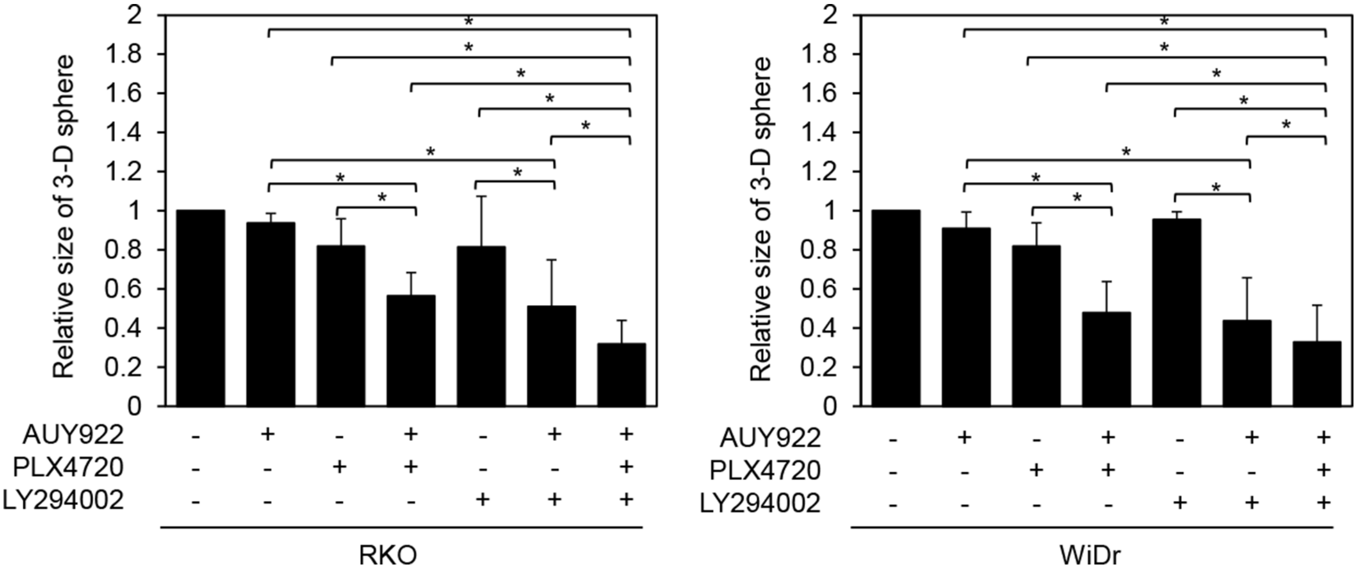

Supplementary Figure S3: The mutant BRAF inhibitor PLX4720 and/or the PI3K inhibitor LY294002 overcome resistance of colon cancer cells grown in 3-dimensional cultures to AUY922. Relative sizes represented by relative diameters of colon cancer cell spheres as shown in Figure 6A. The diameter of the cell sphere treated with vehicle control was arbitrarily designated as 1. Data are mean ± SE, *n* = 3. *$P < 0.05$, Student′s *t*-test.

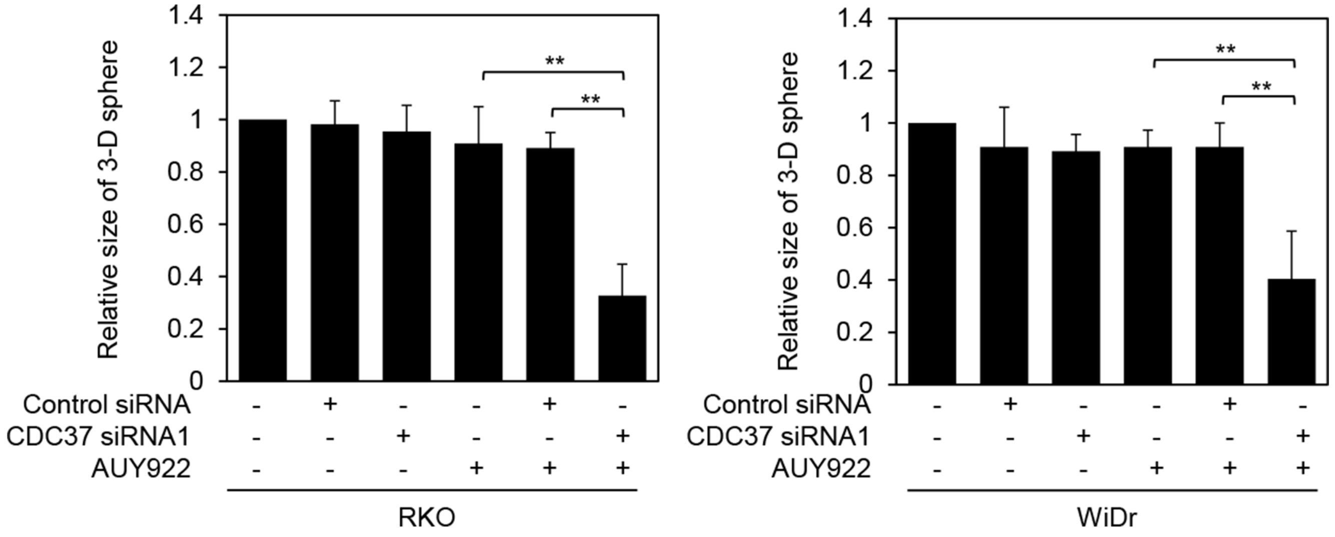

Supplementary Figure S4: Knockdown of CDC37 overcomes resistance of colon cancer cells grown in 3-dimensional cultures to AUY922. Relative sizes represented by relative diameters of colon cancer cell spheres as shown in Figure 6C. The diameter of the cell sphere treated with vehicle control was arbitrarily designated as 1. Data are mean ± SE, $n = 3$. *$P < 0.05$, Student′s t-test.
